# Ventricular Arrhythmia Precipitated by Severe Hypocalcaemia Secondary to Primary Hypoparathyroidism

**DOI:** 10.1155/2019/4851073

**Published:** 2019-04-07

**Authors:** S. Ashwin Reddy

**Affiliations:** Papworth Hospital, Papworth Everard, Cambridge, UK

## Abstract

Hypocalcaemia causes neuromuscular and myocardial symptoms, including QT interval prolongation, and cardiac arrhythmias. Prompt detection and calcium replacement may reverse the pathology, following which the underlying cause should be diagnosed and treated to prevent recurrence. I present the case of a young man presenting with collapse who was found to have sinus rhythm with significant QT interval prolongation on admission electrocardiogram (ECG) associated with profound hypocalcaemia secondary to primary hypoparathyroidism.

## 1. Introduction

The evaluation of blackout in the emergency setting can be challenging due to the myriad potential diagnoses ranging from the benign (such as vasovagal or postural syncope) to the potentially fatal (such as seizure or ventricular tachyarrhythmia). Ventricular arrhythmias may be precipitated by disordered ventricular repolarisation, manifested on the 12-lead electrocardiogram as prolongation of the QT interval beyond 450 ms in males and 470 ms in females when corrected for heart rate. A common but often overlooked cause of QT prolongation are electrolyte disorders, and prompt identification and treatment of these can lead to normalisation of the QT interval.

Primary hypoparathyroidism is a rare condition with a prevalence of 17-22 per 100,000 [1, 2]. Serum hypocalcaemia resulting from this condition can predispose to QT prolongation and present with potentially life-threatening ventricular arrhythmias [3, 4].

## 2. Case Report

A previously healthy 20-year-old Caucasian man was presented to the emergency department following two episodes of loss of consciousness occurring at rest over a fortnight. A witness described collapse followed by shaking of all four limbs but no other epileptiform activity. Spontaneous recovery occurred after a few minutes. The patient denied alcohol or drug use and had an otherwise unremarkable medical history. He had no family history of blackout, collapse, or unexplained sudden death. Physical examination was entirely normal, with specifically no evidence of cardiac failure, no audible murmurs, no tetany, and no facial dysmorphia. He was haemodynamically stable. An initial diagnosis of “seizure” was made in the emergency department pending for further investigations.

**Figure 1:**
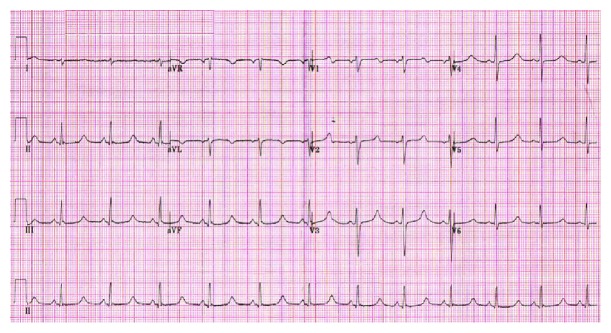
ECG demonstrating sinus rhythm, normal atrioventricular conduction, normal QRS duration, and markedly prolonged QTc.

His ECG demonstrated sinus rhythm with a prolonged corrected QT interval of 588 ms (normal <450) (Figure 1). The PR interval and QRS duration were within normal limits. Admission bloods revealed a corrected calcium of <1.25 mmol/L (normal range 2.2-2.5), phosphate 2.88 mmol/L (0.8-1.4), and alkaline phosphatase (ALP) 172 *μ*/L (35-135). The rest of his bloods, including magnesium, potassium, and renal function, was normal. Endocrine investigations showed a parathyroid hormone (PTH) of <3 ng/mL (14-72), vitamin D 24.1 nmol/L (25-50), and normal thyroid function tests. Echocardiography showed no structural abnormality and preserved ventricular size and function. Computerised tomography (CT) head was also unremarkable.

The patient was admitted to a monitored bed and commenced on intravenous calcium replacement (10 mls 10% calcium gluconate bolus over 30 minutes, followed by infusion of 1% calcium gluconate in one litre of normal saline at a rate of 50 mls/hour). Whilst on the cardiac monitor he was observed to have short episodes of nonsustained polymorphic ventricular tachycardia (VT) which spontaneously resolved; some of these were associated with dizziness but no loss of consciousness. As his serum calcium rose, his ECG normalised and he had no further presyncopal episodes. Once the serum calcium and QTc were within normal limits, he was discharged on oral calcium and vitamin D supplement (Calcichew D3 one tablet twice daily) and 1-alpha-calcidol 1 mcg once daily with early follow-up.

Screening of the patient's mother and younger brother revealed that they too had low levels of PTH and hypocalcaemia but to a less severe degree. His mother's ECG demonstrated a QTc at the upper limit of normal whilst his brother's was within normal limits. The patient was estranged from his father. The family underwent extensive genotyping to identify an underlying cause for their hypoparathyroidism but as yet no clear genetic variant has been identified.

At initial follow-up, the patient's serum calcium and ECG parameters have remained within the normal range, and he has suffered no further syncopal episodes.

## 3. Discussion

Hypocalcaemia predisposes to arrhythmias by affecting both depolarisation and repolarisation of cardiac myocytes. Normally, calcium blocks sodium channels and prevents depolarisation. Decreases in calcium allow increased sodium passage and lowers the depolarisation threshold, causing greater myocardial irritability [5].

Furthermore, hypocalcaemia causes the opening of L-type calcium channels during the repolarisation phase of the cardiac action potential [6], prolonging the QT interval. Late calcium influx and early after-depolarisation then occurs. If the depolarisation threshold is reached, a new action potential can be triggered. As neighbouring myocytes may be at different stages of refractoriness, this may lead to reentrant tachyarrhythmias (namely, torsades de pointes and ventricular fibrillation), thus causing loss of cardiac output and collapse or sudden death. Both the myocardial irritability and QTc prolongation can be reversed by correcting serum calcium.

Aside from QTc prolongation, severe hypocalcaemia may also present with atrioventricular (AV) block, paresthaesia, abdominal cramps, fatigue, altered mood, and tetany. Tetany can sometimes manifest clinically as Chvostek's sign (facial twitching on tapping over the facial nerve just anterior to the ipsilateral ear) or Trousseau's sign (carpal spasm induced by obstructing the brachial artery for 3 minutes with an inflated blood pressure cuff). Seizures and bronchospasm are more severe presentations and constitute an emergency [7, 8].

PTH acts to raise serum calcium by promoting calcium resorption from bone, reducing renal tubular calcium excretion and stimulating the synthesis of 1.25(OH)_2_ vitamin D in the kidney, thus increasing calcium absorption from the small intestine. Hypoparathyroidism thus causes hypocalcaemia [9]. Our patient had undetectably low levels of PTH which, given the serological abnormalities also present in his first degree relatives, is likely due to a presumed though hitherto unidentified genetic condition. PTH replacement therapy is in nascent stages of development: currently, the mainstay of treatment remains oral calcium and vitamin D replacement.

The QTc interval can be abnormally prolonged due to other factors, both acquired and inherited. Acquired causes include drugs (most commonly macrolide and quinolone antibiotics, tricyclic antidepressants and serotonin reuptake inhibitors, antipsychotics, amiodarone, and sotalol), other electrolyte abnormalities (hypokalaemia and hypomagnesaemia), and hypothyroidism. Removal or treatment of the underlying cause will often lead to correction of the QTc interval, often back to normal as in this case.

Inherited long QT syndrome can arise due to a mutation in a gene-encoding ion channels intrinsic to the cardiac action potential and is often inherited in an autosomal dominant manner. The identification of a specific genetic mutation in an index individual allows for cascade screening of relatives, enabling institution of therapy at a presymptomatic stage where appropriate. The mainstay of therapy in inherited long QT syndrome is beta blockade, with second line therapies including flecainide and surgical sympathectomy. In individuals at higher risk of sudden cardiac death, implantation of a transvenous or subcutaneous cardiac defibrillator is indicated. In those untreated who experience syncope or aborted sudden death, the mortality rate at 15 years is around 50%, but this decreases drastically with careful treatment to approximately 1%.

Prolongation of the QTc interval on the 12-lead ECG can be a subtle finding and can be easily missed. This is further complicated by the fact that approximately 2.5% of the normal population will have a prolonged QTc interval. Thus, in young patients presenting with a syncopal episode, particularly in the presence of myoclonic jerking, this can often lead to misdiagnosis of seizures if inadequate care is taken over initial clinical assessment. This not only delays diagnosis and appropriate treatment but also can sometimes lead to the administration of anticonvulsant therapy which has the potential to cause further harm. In this instance, the initial differential diagnosis of seizures was quickly downgraded once the ECG had been performed, and no anticonvulsant treatment was given.

## 4. Conclusion

This case reiterates the importance of considering cardiac arrhythmias as a cause of unexplained syncope with myoclonus and to have a low index of suspicion for considering specifically long QT-associated ventricular tachyarrhythmia. It also strongly demonstrates the importance of accounting for metabolic causes of cardiac rhythm disturbances as these are potentially easily treatable and reversible.
